# Silk Fiber as the Support and Reductant for the Facile Synthesis of Ag–Fe_3_O_4_ Nanocomposites and Its Antibacterial Properties

**DOI:** 10.3390/ma9070501

**Published:** 2016-06-23

**Authors:** Xiaonan Liu, Guangfu Yin, Zao Yi, Tao Duan

**Affiliations:** 1 College of Materials Science and Engineering, Sichuan University, Chengdu 610064, China; liuxiaonan@swust.edu.cn; 2 Joint Laboratory for Extreme Conditions Matter Properties, Southwest University of Science and Technology, Mianyang 621010, China; yizao@swust.edu.cn (Z.Y.); duant@ustc.edu.cn (T.D.)

**Keywords:** Ag–Fe_3_O_4_–silk fiber, antibacterial activity

## Abstract

We report a facile and environmentally friendly approach to prepare Ag–Fe_3_O_4_–silk fiber nanocomposites. The Ag–Fe_3_O_4_–silk fiber acts as: (i) a biocompatible support for the silver nanoparticles; and (ii) a reducing agent for the silver ions. Neither additional reducing agents nor toxic organic solvents were used during the preparation process. The Ag–Fe_3_O_4_–silk fiber nanocomposites can be actuated by a small household magnet and have high antibacterial activities against both *Escherichia coli* and *Staphylococcus aureus*. These nanocomposites could be easily recycled without a decrease in their antibacterial activities due to the synergistic effects between the Ag NPs and Fe_3_O_4_ NPs with large amounts of active sites.

## 1. Introduction

Multi-component nanocomposites include two or more types of nanoparticles and have attracted increasing attention in catalysis, photography, electronic, antibacterial, and optical applications due to their unique functions [1,2]. As a biomedical material, silk fiber plays a key role in the design and synthesis of nanocomposite materials with controllable loading with nanoparticles dispersed on the surface of the chains [2,3,4]. As the most frequently used nanoparticles, magnetic iron oxide (Fe_3_O_4_ and γ-Fe_2_O_3_) nanoparticles and Ag nanoparticles have been employed in the synthesis of advanced nanocomposites. These Fe_3_O_4_ and γ-Fe_2_O_3_ nanoparticles with good biocompatibility and low toxicity can be used for bioseparation, targeted drug delivery, and magnetic resonance imaging [5,6,7,8,9]. However, all of these applications required suitable stabilization of the magnetic nanoparticles to prevent their aggregation and chemical transformation.

Much interest has focused on Ag NPs’ antibacterial activity against a broad spectrum of bacteria and viruses [10,11,12]. In addition, Ag NPs are not yet associated with antibiotic resistance relative to established pharmaceuticals [13]. However, Ag NPs have two primary drawbacks including incomplete removal from the reaction medium and aggregation—this limits them to use in water. The large-scale use of Ag NPs has also been limited by environmental concerns in which Ag NPs can cause serious adverse health effects [14]. Because of their very small size, neither filtration nor centrifugation methods are sufficient to completely remove these Ag NPs from solution. Thus, the combination of magnetic iron oxide NPs and Ag NPs can provide a good alternative because these Ag NPs can be easily separated from the solution via a magnetic field. For nanomaterials, pure NPs are easily assembled into large particles because of the inter-particle dipolar force. This decreases the specific surface area [15]. To inhibit aggregation, NPs can be loaded into various materials such as silica nanospheres [16], graphene oxide [17], silk fiber, etc. [2,3,4]. However, environmentally friendly and sustainable methods are needed to develop renewable energy and green support materials.

Silk fibers are one such example. Silk is one of the most important renewable natural polymers and is an interesting and possible support biomaterial for magnetic NPs and antibacterial Ag NPs because of its good biocompatibility, biodegradation, and non-toxicity [18,19]. Multifunctional silk fibrin fabrics have been made via the chemical assembly of TiO_2_–Ag nanoparticles on the fabric surface. This gives the silk fabric antibacterial activity [20]. Anisotropic Ag NPs have been coated on silk fibers to obtain colorful silk with good antibacterial properties [21].

Here, we describe an easy and environmentally friendly route for the in situ preparation of Fe_3_O_4_ and Ag NPs using silk fiber as a bio-template. In this system, the silk fiber acts as a stabilizer to support and protect the material from agglomeration. To control the number and size of Ag NPs in the shell, we tuned the feeding amount of Ag^+^ ions. The Ag–Fe_3_O_4_–silk fiber composites have excellent antibacterial ability against *E. coli* and *S. aureus*. These results indicate that Ag–Fe_3_O_4_–silk fiber composites may have a potential application as disinfectants for water treatment.

## 2. Materials and Methods

### 2.1. Materials

All chemicals including silver nitrate (AgNO_3_), silk fiber, and ferric chloride hexahydrate (FeCl_3_·6H_2_O) were supplied by Kelong Chemical Reagent Co., Ltd. (Chengdu, China) and used without further purification.

### 2.2. Preparation of the Fe_3_O_4_–Silk Fiber

The Fe_3_O_4_–silk fiber composites were prepared through an improved one-step hydrothermal method [22,23]. Typically, FeCl_3_·6H_2_O (0.1622 g), NaHCO_3_ (0.756 g), and vitamin C (0.0294 g) were all dissolved in deionized water (40 mL) under magnetic stirring to form a homogeneous solution. Then, 1-g silk fibers were immersed in the solution. Finally, the resulting solution was transferred to a Teflon-lined stainless-steel autoclave (50-mL capacity). The autoclave was heated at 150 °C for 4 h and allowed to cool to room temperature. The resulting black products were washed with deionized water and ethanol several times and then dried in a vacuum for 12 h.

### 2.3. Preparation of the Ag–Fe_3_O_4_–Silk Fiber and Ag–Silk Fiber

The Ag–Fe_3_O_4_–silk fiber composites were prepared through an improved electro-less plating approach [24]. At room temperature, 2 mL of AgNO_3_ solution (0.01 M, 0.02 M, and 0.03 M) was added into a conical bottle followed by the dropwise addition of 10 mL of aqueous triethanolamine (TEA) solution (the complex reducing agent). Some brown sediment settled to the bottom of the bottle during the reaction. This sediment then completely dissolved, indicating that [Ag(TEA)^2^]^+^ was generated. After that, deionized water was added to obtain a total volume of 100 mL. The Ag–Fe_3_O_4_–silk fiber composite or silk fibers were immersed in the reaction solution for about 1 h. Then, the Ag–Fe_3_O_4_–silk fiber composites were washed with deionized water to remove the untreated AgNO_3_. The as-prepared Ag–Fe_3_O_4_–silk fiber samples with different AgNO_3_ concentrations were signed as Ag–Fe_3_O_4_–silk fibers (1#, 2#, and 3#).

### 2.4. Antimicrobial Activity of the Ag–Fe_3_O_4_–Silk Fiber and Ag–Silk Fiber

The sterile Ag–Fe_3_O_4_–silk fiber, Ag–Silk fiber, Fe_3_O_4_–silk fiber, and control silk fiber were individually prepared and dried at 30 °C. Separate tubes of Luria-Bertani (LB) Broth were inoculated with overnight cultures of two bacterial strains *Escherichia coli* (*E. coli*, ATCC 25922, a Gram-negative bacteria) and *Staphylococcus aureus* (*S. aureus*, ATCC 25923, a Gram-positive bacteria) to give an initial bacterial density of 10^8^ CFU/mL. This was over laid onto agarose medium plates.

Tests were performed in triplicates using the Ag–Fe_3_O_4_–silk fiber, Ag–Silk fiber disks as possible antibacterial materials, the silk fiber as the negative control, and Kanamycin as the positive control. The Petri plates were incubated at 37 °C for 24 h, and the inhibition zone formed around each disc was later measured via photography.

### 2.5. Characterization Methods

The phase compositions of the samples were confirmed by X-ray diffraction (XRD, X’Pert PRO, PANalytical B.V, Almelo, The Netherlands) with Cu-Kα radiation operating at 2.2 kW, and the step size was 0.0167111. The microstructure of the samples was characterized with a scanning electron microscope (SEM, Ultra 55, Carl Zeiss AG, Heidenheim, Germany), and the element constitution and element mapping of the samples were characterized with the FE-SEM (JSE-7500F, JEOL, Tokyo, Japan) equipped with an EDS accessory (INCA250 X-MAX 50 system, Oxford Instruments, Oxford, UK). The static magnetic properties of the samples were determined using a vibration sample magnetometer (BKT-4500Z, Zetianweiye, Beijing, China) at room temperature. 

### 2.6. Quantification of Relesased Ag^+^ by ICP-MS

In order to determine whether the antimicrobial activity on samples was possibly affected by the nano Ag (or Ag^+^) released to the nutrient media, the media were analyzed for the presence of Ag. The concentration of Ag was measured by an inductively coupled plasma mass spectrometer (ICP-MS, iCAP6500, Thermo Fisher Scientific, Renfrew, UK).

## 3. Results and Discussion

**Figure 1:**
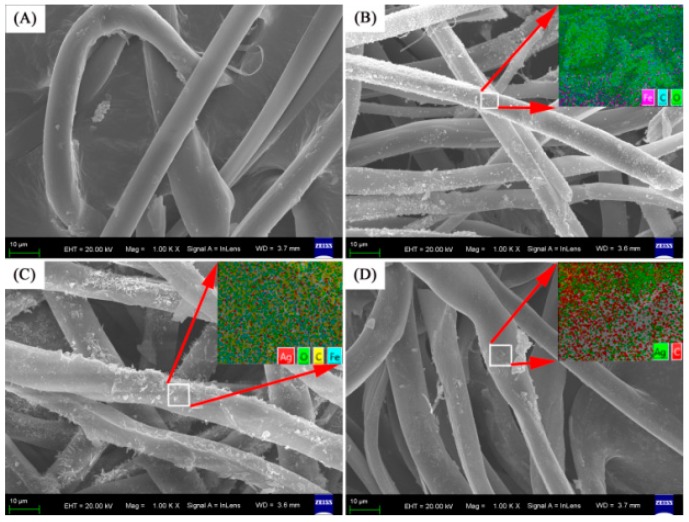

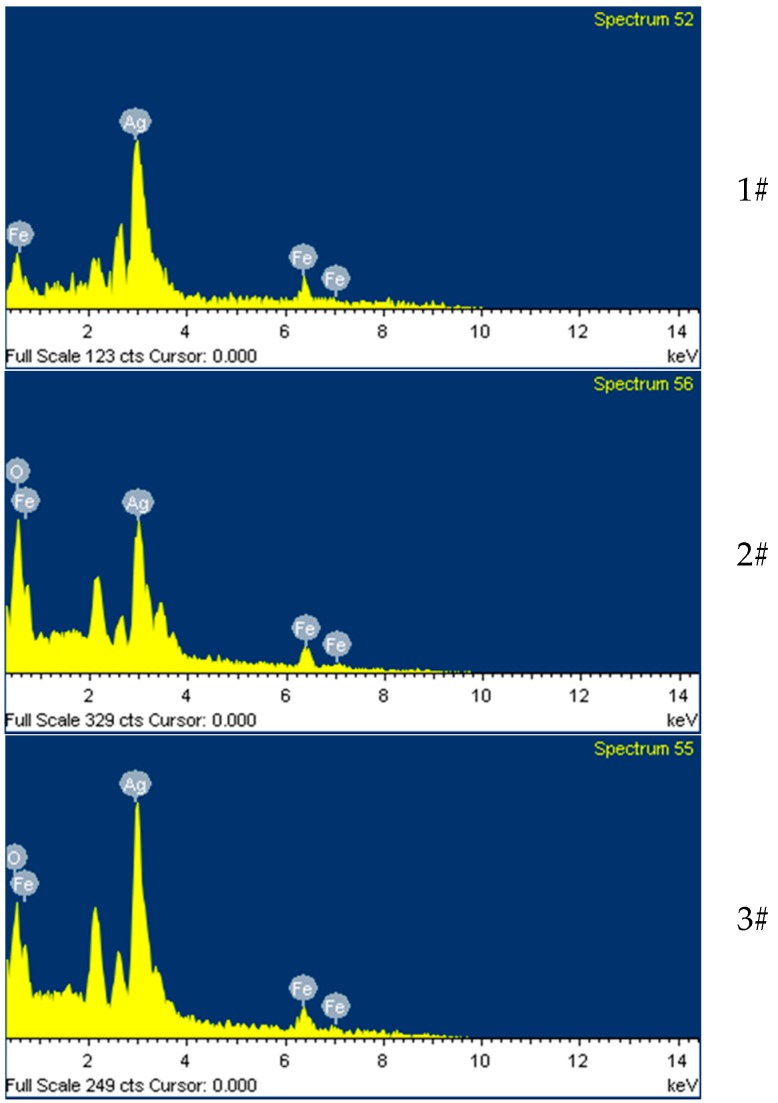
Typical scanning electron microscopy (SEM) images of the samples: (**A**) silk fiber; (**B**) Fe_3_O_4_–silk fiber; (**C**) Ag–Fe_3_O_4_–silk fiber (2#); and (**D**) Ag–Silk fiber. EDX spectra of Ag–Fe_3_O_4_–silk fibers (1#–3#).

SEM images were captured to explore the surface morphologies of the unmodified and modified silk fibers (Figure 1). The degummed silk fibers show very smooth surfaces. This suggests the successful removal of sericin from raw silk (Figure 1A). After this hydrothermal reaction, some small dots appeared on the silk surfaces (Figure 1B) indicating the efficient in situ synthesis of Fe_3_O_4_ NPs. The inset of Figure 1B shows the EDX elemental maps of the Fe_3_O_4_–silk fiber composite. The uniform distribution of Fe_3_O_4_ NPs in the composite was confirmed from the maps. As the Fe_3_O_4_–silk fiber composite formed in the AgNO_3_ solution, the density of the dots gradually increases, and the dots aggregate to form clusters (Figure 1C and Figure S1). The inset of Figure 1C shows the EDX elemental maps of the Ag–Fe_3_O_4_–silk fiber composite. The uniform distribution of Ag NPs and Fe_3_O_4_ NPs in the composite was confirmed via these maps. The Ag NPs were synthesized evenly on the fiber surface (Figure 1D). No bulk aggregates were present in the field of view. The EDX elemental map confirms that the Ag is present on the Ag–silk fiber composite surfaces. According to the EDX data, the weight percent values of Ag were 0.58% (1#), 1.14% (2#), and 1.75% (3#).

**Figure 2:**
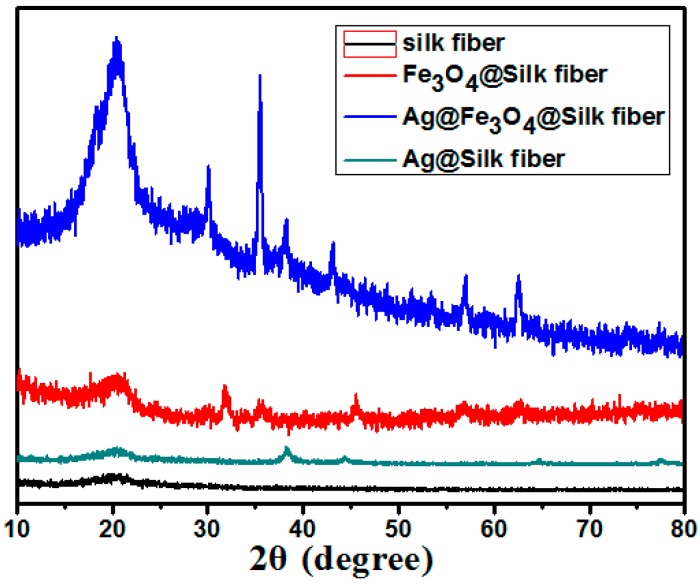
The X-ray diffraction (XRD) patterns of the silk fibers, Fe_3_O_4_–silk fibers, Ag–Fe_3_O_4_–silk fibers (2#) and Ag–Silk fibers.

The XRD patterns are shown in Figure 2. This depicts the crystal structure of the silk fiber, Fe_3_O_4_ NPs, and Ag NPs. The iron oxide phase in the Fe_3_O_4_ NPs can be verified from the diffraction peaks at 30.0°, 35.3°, 42.8°, 56.9°, and 62.5°. They correspond to the (220), (311), (400), (511), and (440) planes of the face-centered cubic (fcc) structure of Fe_3_O_4_ (JCPDS No. 87-2334), respectively. This indicates the successful synthesis of the Fe_3_O_4_ NPs [25]. Furthermore, the Fe_3_O_4_/Ag–Silk fibers display four major peaks at 38.2°, 44.4°, 64.6°, and 77.5° corresponding to the (111), (200), (220), and (311) crystal planes, respectively. This indicates the fcc structure of the Ag NPs (JCPDS No. 87-0720) [26].

**Figure 3:**
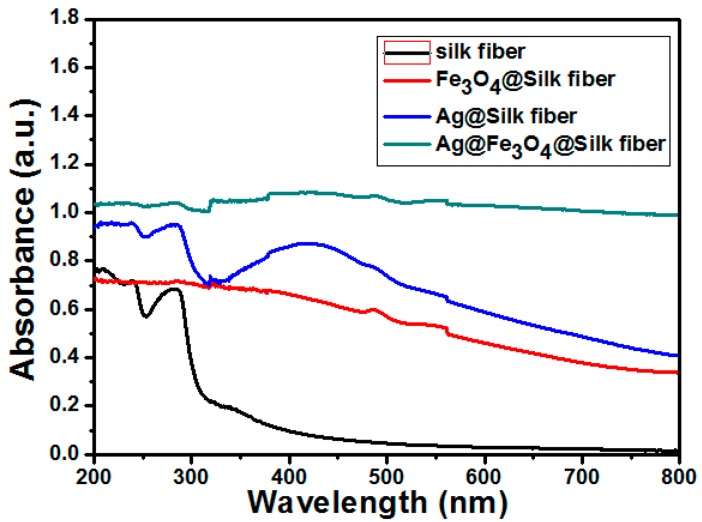
UV-vis spectrum of the silk fibers, Fe_3_O_4_–silk fibers, Ag–Fe_3_O_4_–silk fibers (2#), and Ag–Silk fibers (2#).

One of the most important and unique properties of nanostructured precious metals is their localized surface plasmon resonance (LSPR). This appears when the size of the nanostructures becomes comparable to or smaller than the mean free path of the conducting electrons in the metal. Hence, the UV-vis adsorption spectrum of the silk fibers, Fe_3_O_4_–silk fibers, Ag–Fe_3_O_4_–silk fibers (2#), and Ag–Silk fibers were measured, as showing in Figure 3. The surface plasmon resonance peak of the Ag–Fe_3_O_4_–silk fibers (2#) was wide relative to the spectrum of the Fe_3_O_4_–silk fibers and Ag–Silk fibers. This results from the electron transfer across the Ag–Fe_3_O_4_ interface [27].

**Figure 4:**
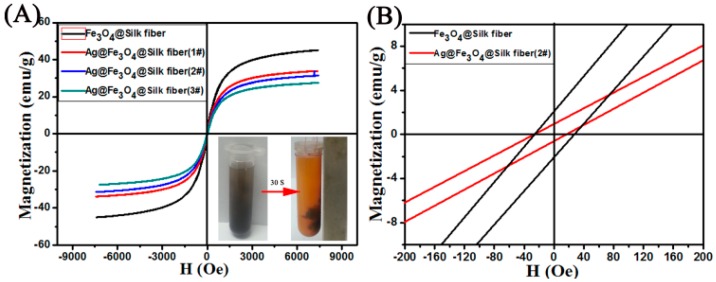
(**A**) Room temperature magnetic hysteresis loops for Fe_3_O_4_–silk fibers, Ag–Fe_3_O_4_–silk fibers (1#), Ag–Fe_3_O_4_–silk fibers (2#), and Ag–Fe_3_O_4_–silk fibers (3#). Response of the Ag–Fe_3_O_4_–silk fibers (2#) to an external magnet (inset of Figure 4A); (**B**) Magnification of the magnetic hysteresis loops in the region of the most rapid change with changing applied field intensity (Fe_3_O_4_–silk fibers and Ag–Fe_3_O_4_–silk fibers (2#)).

Next, the magnetic properties of the Fe_3_O_4_–silk fibers and the Ag–Fe_3_O_4_–silk fibers (1#–3#) were investigated because the superparamagnetic properties of these materials are critical to ensuring their proper applications. The saturation magnetization (Ms) of the Fe_3_O_4_–silk fibers, Ag–Fe_3_O_4_–silk fibers (1#), Ag–Fe_3_O_4_–silk fibers (2#), and Ag–Fe_3_O_4_–silk fibers (3#) are 45.06 emu·g^−1^, 33.83 emu·g^−1^, 31.38 emu·g^−1^, and 27.41 emu·g^−1^, respectively (Figure 4A). A lower Ms was observed for the Ag–Fe_3_O_4_–silk fibers than the Fe_3_O_4_–silk fibers. This reduction in the nanocomposites originates from the interactions between the Fe_3_O_4_ and Ag NPs as well as the presence of the non-magnetic Ag NPs [28]. Further confirmation and examination of the Ms of the hybrids with different amounts of Ag NPs is currently under investigation. The remanence (Mr) and coercivity (Hc) of the Fe_3_O_4_–silk fibers are 1.1 emu·g^−1^ and 13.98 Oe. For the Ag–Fe_3_O_4_–silk fibers (2#), they are 1.3 emu·g^−1^ and 17.04 Oe, respectively (Figure 4B). Due to this small remanence and coercivity, the Fe_3_O_4_–silk fibers and Ag–Fe_3_O_4_–silk fibers (2#) exhibit superparamagnetic behavior.

Of note, the Ms of the Ag–Fe_3_O_4_–silk fibers (2#) prepared in this study were much higher than that of the magnetic antibacterial nanocomposites (18 emu·g^−1^) prepared by Sureshkumar et al. [29]. The higher Ms ensured a better magnetic response of the magnetic nanocomposites toward an external magnetic field. The strong magnetic response of both samples is also demonstrated by the photographs in Figure 4A. These results indicate that the Ag–Fe_3_O_4_–silk fibers possess good magnetic properties. This ensures a good magnetic response in the application of magnetic field-assisted separation of the Ag NPs.

**Figure 5:**
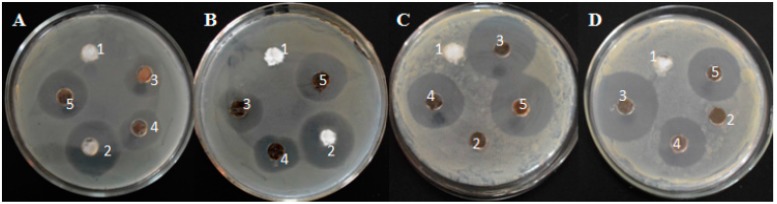
Representative images containing the inhibition zone of *E. coli* with 10^8^ CFU/mL (**A**,**C**) and *S. aureus* with 10^8^ CFU/mL (**B**,**D**). Samples A1–5 and B1–5 are silk fibers, silk fibers with Kanamycin, Ag–Fe_3_O_4_–silk fibers (3#), Ag–Fe_3_O_4_–silk fibers (2#), and Ag–Fe_3_O_4_–silk fibers (1#), respectively. Samples C1–5 and D1–5 are silk fibers, Fe_3_O_4_–silk fibers, silk fibers with Kanamycin, Ag–Silk fibers, and Ag–Fe_3_O_4_–silk fibers (1#), respectively.

We investigated the antimicrobial activity of the Ag–Fe_3_O_4_–silk fibers against *E. coli* and *S. aureus* using the disk diffusion method. Figure 5 shows that the Ag–Fe_3_O_4_–silk fibers have good antibacterial effects for both bacterial strains. Furthermore, the sample with the smaller mass proportion of Ag has a slightly narrower zone, whereas the sample Ag–Fe_3_O_4_–silk fibers (1#) (33.83 emu·g^−1^) has wider inhibition zones. In addition, the Ag–Fe_3_O_4_–silk fibers (1#) have a marginally stronger antibacterial activity than the Ag–silk fibers. The antibacterial activity of the Ag–Fe_3_O_4_–silk fibers is similar to that of the silver nanoparticle-hydroxyapatite composites. This is attributed to the release of Ag^+^ into the media [30]. Silver can bind to the bacterial cell wall and cell membrane to inhibit respiration and DNA replication [31]. To further evaluate the effect of Ag^+^ on antimicrobial activity, the amount of Ag^+^ released into the nutrient media from the Ag–Fe_3_O_4_–silk fiber nanocomposites was measured by ICP-MS. The obtained results are summarized in Table 1.

## 4. Conclusions

In conclusion, we explored a facile and environmentally friendly method of preparing Ag–Fe_3_O_4_–silk fiber nanocomposites consisting of magnetic NPs, Ag NPs, and silk fibers. It acted as a stabilizer to support the protecting agent against agglomeration. The superior magnetic properties of the Ag–Fe_3_O_4_–silk fiber nanocomposites ensured a good magnetic response for the separation process. The preliminary antibacterial assays indicated that the Ag–Fe_3_O_4_–silk fibers possess excellent antibacterial ability against *E. coli* and *S. aureus*. This might be due to the synergistic effects between AgNPs and Fe_3_O_4_NPs affixed to the one-dimensional (1D) silk nano- or microfibers. Given these advantages, we believe that these Ag–Fe_3_O_4_–silk fiber nanocomposites can be broadly used as a recyclable anti-bacterial agent in medical and environmental applications.

## Figures and Tables

**Table 1:** The amount of Ag released into the nutrient media from the Ag–Fe_3_O_4_–silk fiber nanocomposites, as measured by ICP-MS.

Materials	Amount of Ag in Ion State (*X*) (μg/mL)	Total Amount of Ag (*Y*) (μg/mL)	*Y* Minus *X* (μg/mL)
silk fibers	0	0	0
Ag–Fe_3_O_4_–silk fibers (1#)	3.2	7.6	4.4
Ag–Fe_3_O_4_–silk fibers (2#)	4.7	10.2	5.5
Ag–Fe_3_O_4_–silk fibers (3#)	5.3	14.8	9.5

## Supplementary Materials

The following are available online at www.mdpi.com/1996-1944/9/7/501/s1. Figure S1: SEM of Ag@Fe_3_O_4_@Silk fiber (2#).
