# Supplementary material for: Silk Fiber as the Support and Reductant for the Facile Synthesis of Ag–Fe3O4 Nanocomposites and Its Antibacterial Properties
Source: Materials (Basel). 2016 Jun 23;9(7):501. doi: 10.3390/ma9070501 (PMC5456862; doi:10.3390/ma9070501)
Supplement: Supplementary file 1 [file materials-09-00501-s001.pdf]

Supplementary Materials: Silk Fiber as the Support and Reductant for the Facile Synthesis of Ag–Fe_3O_4 Nanocomposites and Its Antibacterial Properties

Xiaonan Liu, Guangfu Yin, Zao Yi and Tao Duan

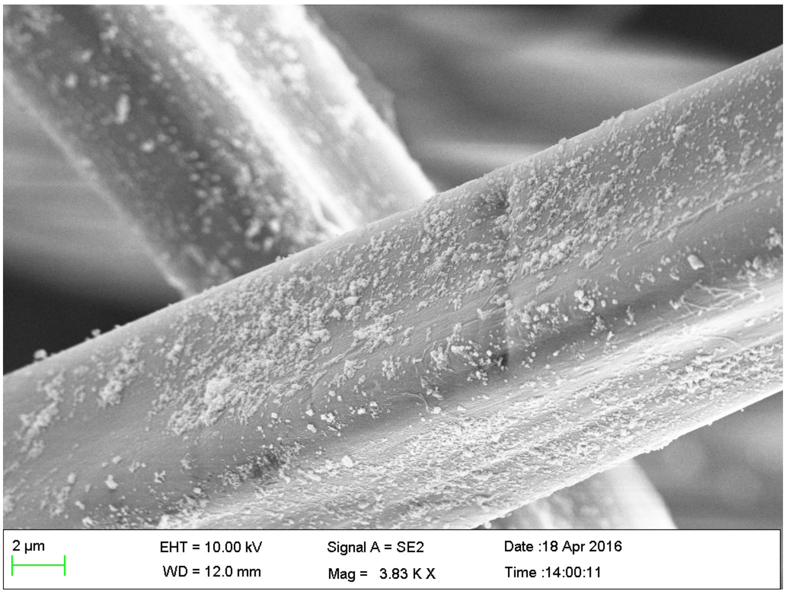

Figure S1. SEM of Ag–Fe_3O_4–Silk fiber (2#).
